# Perspectives on the Measles, Mumps and Rubella Vaccination among Somali Mothers in Stockholm

**DOI:** 10.3390/ijerph15112428

**Published:** 2018-11-01

**Authors:** Asha Jama, Mona Ali, Ann Lindstrand, Robb Butler, Asli Kulane

**Affiliations:** 1 Unit for Vaccination Programmes, The Public Health Agency of Sweden, SE-171 82 Solna, Sweden; asha.jama@folkhalsomyndigheten.se (A.J.); mona.m.ali1987@gmail.com (M.A.); Ann.Lindstrand@folkhalsomyndigheten.se (A.L.); 2 Division of Communicable Diseases and Health Security, WHO Regional Office for Europe, DK-2100 Copenhagen, Denmark; butlerr@who.int; 3 Equity and Health Policy Research Group, Department of Public Health Sciences, Karolinska Institute, Tomtebodavägen 18, 171 77 Stockholm, Sweden

**Keywords:** MMR vaccination, measles, vaccine hesitancy, autism, Rinkeby, Tensta

## Abstract

*Background*: Vaccination hesitancy and skepticism among parents hinders progress in achieving full vaccination coverage. Swedish measles, mumps and rubella (MMR) vaccine coverage is high however some areas with low vaccination coverage risk outbreaks. This study aimed to explore factors influencing the decision of Somali parents living in the Rinkeby and Tensta districts of Stockholm, Sweden, on whether or not to vaccinate their children with the measles, mumps and rubella (MMR) vaccine. *Method*: Participants were 13 mothers of at least one child aged 18 months to 5 years, who were recruited using snowball sampling. In-depth interviews were conducted in Somali and Swedish languages and the data generated was analysed using qualitative content analysis. Both written and verbal informed consent were obtained from participants. *Results*: Seven of the mothers had not vaccinated their youngest child at the time of the study and decided to postpone the vaccination until their child became older (delayers). The other six mothers had vaccinated their child for MMR at the appointed time (timely vaccinators). The analysis of the data revealed two main themes: (1) barriers to vaccinate on time, included issues surrounding fear of the child not speaking and unpleasant encounters with nurses and (2) facilitating factors to vaccinate on time, included heeding vaccinating parents’ advice, trust in nurses and trust in God. The mothers who had vaccinated their children had a positive impact in influencing other mothers to also vaccinate. *Conclusions*: Fear, based on the perceived risk that vaccination will lead to autism, among Somali mothers in Tensta and Rinkeby is evident and influenced by the opinions of friends and relatives. Child Healthcare Center nurses are important in the decision-making process regarding acceptance of MMR vaccination. There is a need to address mothers’ concerns regarding vaccine safety while improving the approach of nurses as they address these concerns.

## 1. Introduction

Vaccines are one of the safest and most effective medical interventions in history; yet there has been a surge in concerns related to vaccination, leading parents to become hesitant to immunize their children [1]. Vaccine hesitancy refers to delay in acceptance or refusal of vaccines despite availability of vaccination services [2]. Vaccine hesitancy is a global challenge to the achievement of full vaccination coverage [3].

The most commonly reported reason for vaccine hesitancy is concern regarding the safety of one or more vaccines [4], and most common is the fear of presumed side-effects of the MMR vaccine [5]. Autism, immune system overload, and other presumed adverse reactions have often been cited [6]. Some parents do not recognize the full benefits of childhood immunizations, and can be especially skeptical of routine schedules that call for multiple immunizations given simultaneously on a strict timeline. Other reasons for not vaccinating include belief that a child is too young or too sick to receive a vaccine. Parents who accept vaccines are generally confident that immunization is a proven method to prevent disease [7].

Vaccine hesitancy varies greatly with context and across time, place and vaccines [8,9]. According to the Strategic Advisory Group of Experts (SAGE) working group matrix for vaccination hesitancy, several individual and contextual issues play a role in addition to the vaccine-specific issues [10]. The impact of social networks can also not be ignored [11]. These networks form a cohort of influencers that drive opinions on immunization [12].

Growing hesitancy related to MMR vaccination existed even among parents who have vaccinated their children [13]. Studies have shown that even parents who have previously consented to vaccination can be negatively influenced by rumours and ongoing debates [14]. 

MMR vaccine hesitancy has been found to be more prevalent among some faith based group or ethnic minorities and this has increased interest in identifying prevailing attitudes toward MMR vaccination [15,16,17]. The ethnic minorities include Somali parents living in Birmingham (UK) who perceived that there is a link between autism and MMR vaccine [18]. Similarly the Somali community in Minnesota (USA) have low MMR vaccine coverage and attribute it to concerns regarding the higher than average prevalence of autism spectrum disorder in the community [19].

In Sweden, MMR vaccine is offered at Child Healthcare Center at age 18 months and at age 6–8 years in schools. Overall, MMR vaccination coverage in Sweden at age 2 years is 97.5% [20] however several communities have vaccine coverage below 95%, the level needed to maintain herd immunity for measles. Child Health Centres (CHCs) in the Rinkeby and Tensta districts of Stockholm have reported MMR coverage of 71.5% and 69.7% respectively in 2012 [20]. The two districts have high percentage of residents with foreign background and approximately 30% with a Somali origin (20). The vaccination coverage has remained low since the late 1990s, when Wakefield published the later refuted article on a presumed link between autism and the MMR vaccine [20,21]. The current study aimed to identify factors influencing the decision of Somali parents living in Rinkeby and Tensta on whether or not to vaccinate their children with the MMR vaccine.

## 2. Materials and Methods

### 2.1. Study Design and Setting

This study is a part of larger project using the tailoring immunization programmes (TIP) methodology developed by the World Health Organisation (WHO) to find motivators and barriers to vaccination [22]. The study design is explorative with inductive qualitative approach. In depth interviews were used [23]. The study was conducted in Rinkeby and Tensta, districts located in the northwest part of Stockholm with a high percentage of residents with foreign backgrounds. The population in Rinkeby district was 16,047 in 2013, including 1638 children under five years (8.9%). The population of Tensta was 18,866, including 1673 children under five years (10.2%). An estimated 30% of this population is of Somali origin. This sub-population was chosen for the study based on concerns expressed by health workers regarding vaccine hesitancy specifically among Somali parents, and low MMR vaccination coverage in these districts [20].

### 2.2. Participants

Parents of children aged 18 months to 5 years were recruited for this study through different routes. A community stakeholders group consisting of local Somali nongovernmental organizations (NGOs), parent groups and mosque leaders assisted in the recruitment process. The leaders of these community groups were approached to inform potential participants, who in turn invited others who met the inclusion criteria in a process of snowball sampling. In addition, written invitations were available and posters pinned at the CHCs, but no participants were recruited through this channel. Thirteen mothers volunteered to be interviewed. Recruitment was stopped after 13 participants when no more new information was coming from the interviews [24]. All of the invited fathers declined. One father was present during the interview with his wife but declined to comment.

### 2.3. Data Collection

In-depth interviews (IDIs) were used to collect data because interviewing is the best-suited method to gain insight into parents’ experiences and perspectives in deciding about vaccination for their children [24]. All interviews were conducted during June to September 2013. The participants chose the venues for the interviews. Most interviews took place at the interviewee’s home, one took place at the CHC and three were conducted by phone. In most cases, only the interviewer, the note taker, the mother and her child were present, ensuring that confidentiality was maintained. The interviewer used probing and question rephrasing techniques to clarify questions and obtain details from the participants. The interviews were conducted by the first two authors (AJ & MA) and the last author (AK) in either Swedish or Somali, depending on the participant’s choice. Each individual interview lasted around 30 to 60 min, with the exception of one that lasted 15 min.

### 2.4. Data Analysis and Interpretation

The interviews were transcribed verbatim and the researchers read all of the obtained data. The results were interpreted using a qualitative content analysis method as described by Graneheim and Lundman [25]. This method helps researchers to interpret and understand both the manifest and latent or hidden meanings. The researchers developed codes for the meaning units and also coded data within the same content area to identify differences and similarities. Codes and similarities were then put together in a category to show manifest meaning, from which themes emerged. Peer debriefing was conducted, in which the preliminary results were shared with the rest of the team and thereafter member checking was conducted to ensure credibility [26].

### 2.5. Research Ethics

The study was approved by the Regional Ethics Committee, Stockholm, Sweden (Dnr 2013/678–31/3). All participants were given information about the study and its objectives prior to giving their verbal consent to participate. Verbal consent was chosen for anonymity of the participants and was was approved by the ethics board. The information provided to participants covered the aim of the study, voluntariness, confidentiality and the option to discontinue participation at any time during the study. The participants were also provided with the contact information of the responsible researchers. All collected data was coded to ensure anonymity and kept within the research group’s locked facilities.

## 3. Results

The 13 mothers were aged 25 to 42 years. Two of them had two children, six had three children, one had four children, two had five children, one had six children and one had seven children.

Seven of the mothers had not vaccinated their youngest child within the selected age group for MMR and had decided to postpone the first dose of this vaccination until their child became older (delayers). The remaining six mothers had vaccinated their child for MMR at the appointed time (timely vaccinators).

The analysis of the data revealed two main themes: (1) barriers to vaccinate on time, consisting of two categories (*issues surrounding fear of child not speaking* and *unpleasant encounters with nurses*) and (2) facilitating factors to vaccinate on time, consisting of three categories *heeding vaccinating parents’ advice*, *trust in nurses* and *trust in God*.

### 3.1. Barriers to Vaccinate on Time

#### 3.1.1. Issues Surrounding Fear of Child Not Speaking

Parents who chose to delay their child’s vaccination had experienced peer pressure from other parents in their social network, including parents they did not known personally. This peer pressure created a perception among the delaying mothers that they would put their children’s ability to speak at risk if they accepted the vaccine. The parents met these people in many settings, such as gatherings where women meet to listen to religious leaders and other social events, but most often at the CHCs as illustrated in this quote:

*“No I don’t meet anyone [them] here in the playground, it’s the CHC centre where we meet”*.

Delayer 6

It is at the CHCs where mothers obtain advice about their children’s health however the mothers expressed that they often sought additional advice from other mothers before the routine child care visits. They made phone calls to mothers whom they perceived to have more experience in raising children and had older children who had gone through these routine programmes.

*“I ask other mothers who have had children and have more experience”*.

Delayer 3

If they spoke to other mothers who had delayed vaccination, this fueled their suspicion that vaccination at 18 months was not advisable. The messages and opinions received during these conversations were given serious consideration by mothers who eventually chose to delay vaccination.

*“It was an aunt, an older woman whose child had received it[vaccine] and then became ill of it and since then I know about it. It has happened, so that is why one thinks, no thanks”*.

Delayer 11

Some parents believe that vaccinations prevent diseases, but they delay vaccination beyond the age of 18 months to avoid the perceived risk of vaccine side effects, especially related to speech development. They preferred to wait until the child could speak fluently as illustrated below.

*“I would like to get more information about this 18 months vaccine. It is the one people say makes “children stop talking”. I waited to see if this child stopped talking by himself. I wanted to see him start talking”*.

Delayer 6

Vaccine hesitant parents in the area who are worried about the MMR vaccine convey their fears to parents who want to vaccinate their children on time. This was reported to create peer pressure, and mothers who have vaccinated their children on time may begin to doubt their earlier decisions.

*“I would like to add that parents who are worried forward their worries to parents who want to vaccinate. It happens very often”*.

Timely vaccinator 2

Uncertainty about vaccine safety fueled by these rumours makes some mothers reluctant to have their children vaccinated for MMR, and leads them to delay the vaccination.

*“One becomes afraid as a parent… when there is so much talk about how bad it [the MMR vaccine] is. Of course they say it has nothing to do with the vaccination and it’s proven, but you still don’t know. One doesn’t want to have a bad conscience later in life as a parent… That’s how I reasoned, but you never know what’s bad or good really”*.

Delayer 13

The delaying mothers mentioned the fear of other development disorders and that they did not want to risk their children developing any type of mental disorders by giving the MMR vaccine. Some did not understand the reasons for vaccinating against MMR.

*“Why should one take it if your child is not in need of it, if she doesn’t take it nothing happens but if she does there is a risk”*.

Delayer 11

Others doubted the effectiveness of the vaccine and used this reason to delay or avoid the decision.

*“I’m worried because one cannot say that all vaccines work for all children. I believe that maybe some react differently than others do. In special cases, not in the majority of course but I believe that some get ill from it. I really believe it has to do with something in that child’s genes. One never knows if one’s child is like that and that is the fear I believe. I don’t know how they will react, if they will react positively to it”*.

Delayer 7

Parents’ negative attitudes toward vaccinations are particularly against the MMR vaccine. The participants said that other mothers told them not to vaccinate for MMR, however it was fine to receive other vaccines because they prevent deadly diseases and are safe.

*“You can take the other vaccines. They are for the six diseases for example against polio, pneumonia, cold and also malaria”*.

Delayer 1

#### 3.1.2. Unpleasant Encounters with Nurses

Some mothers who had had negative experiences related to how they were received at the CHC were compelled to delay vaccination. The negative experiences, which had even caused some to change centres to those outside their areas of residence, were a driving force for these mothers’ hesitancy. These experiences included how the mothers were greeted at the CHCs by the nurses and how they were addressed with information in regard to vaccination.

*“It was bad information I think and also this nurse, she was tired of the work and maybe had worked there too long and seen too many faces. I felt the encounter was very boring. It was a burden for me to go there so I stopped going”*.

Delayer 11

The mothers further shared nurses’ perceptions of Somali parents who had not vaccinated their children. The mothers felt they were judged, had no chance of explaining their worries, and were denied the opportunity to get information about the vaccine.

*“When I went there she said that all Somalis avoided this vaccine and why don’t you want to vaccinate. She already had her own answers. She didn’t want to know my reason. I understood that she had her own answers and I from Somalia am already being judged. “Yes yes, I know all of you don’t want this vaccine and avoid it and you think you can get autism”. I just was quiet and listened. Do you think so? I asked. “ Yes, we believe so”, she responded. I replied “Okay I am the mother of [ name of the child] and I am going to wait [to vaccinate], then it was only goodbye”*.

Delayer 13

This feeling of resentment and the perceived lack of interest in what they had to say drove these mothers away from the vaccination encounter, even though they acknowledged that the nurses appeared to be overwhelmed by their work.

*“The staff are a little stressed, because they have a staff shortage and they have had it forever because [name of centre] is responsible for the whole of [ name of area]. So the staff are not many, and it is not enough I think. But they do what they can”*. Delayer 12

### 3.2. Facilitating Factors to Vaccinate on Time

#### 3.2.1. Heeding Vaccinating Parents’ Advice

All parents shared that they had friends and relatives who told them about their perceptions regarding autism being caused by the MMR vaccine. Even the parents who decided to vaccinate on time said everyone around them was talking about autism; however, those who got positive feedback from their friends went ahead to vaccinate their children.

*“…One of them is my best friend who is my only friend who came to this country before me and knows the language, works and knows much about health issues. She has one son and vaccinated her son”*.

Timely vaccinator 2

These mothers were able to dismiss claims about the perceived negative side effects of the MMR vaccine because of the positive influence of their colleagues or friends who were vaccinating on time.

*“Sometimes there are places where a lot of people gather for religious events where mothers with young children can talk to each other. They usually ask how old is your child? Have you vaccinated him for one and a half year old vaccine? Yes. Has he not stopped talking? No, I answer.[Laughter]. I don’t think that is true”*.

Timely vaccinator 4

#### 3.2.2. Trust in Nurses

Parents who had more trust in the nurses vaccinated on time, asked more questions and believed the answers they received from the nurses.

*“Now I ask advice from the nurse. Before it was the Somali talk I used to listen to… Nowadays I take what the nurse says”*.

Timely vaccinator 1

Some timely vaccinating mothers discredited the rumours and had their children vaccinated on time following positive discussions with the nurses. A couple of mothers who did not vaccinate their older children for the MMR vaccine but later changed their minds and vaccinated their younger children said that the nurse had an impact on their decision to vaccine on time.

*“I have received all information, but all this talk about the vaccine is not true she said. And I have observed and there are no differences that I have noticed on children either”*.

Timely vaccinator 5

These timely vaccinators strongly believed that the healthcare providers would never do anything to harm their children but rather had an interest in keeping their children healthy. They put the health of their children first and entrusted the nurses with the duty of protecting their children’s health.

*“I have never seen that someone who has been vaccinated stops talking. I often say that these are doctors and healthcare workers, I don’t believe they would do anything to harm children”*.

Timely vaccinator 4

#### 3.2.3. Trust in God

All mothers who vaccinated for MMR on time had confidence in their decision to vaccinate their children. Their motivation was that they trusted God and believed anything that happened to their children was according to the will of God. They shared that if their child became ill then it was the will of God and no one could do anything to prevent it.

*“I believe that God has given you your child yesterday and can as quickly give him something tomorrow. He can give him something after twenty years or when he is little. One should believe in God, that is very important”*.

Timely vaccinator 2

## 4. Discussion

This study found that concerns about MMR vaccine is evident among the Somali mothers in the Rinkeby and Tensta districts in Stockholm, Sweden. These results echo the opinions expressed by Somali women residing in the United Kingdom [18]. The strong component of mistrust and fear of MMR vaccine side effects was illustrated in both studies. The perceived side effect feared most is that the child may stop speaking. Other previous studies in this setting in Sweden [21], mothers skipped information meetings at CHCs and relied on receiving that information from the one or few mothers who did attend, relying more on mother’s advice than nurses’ advice.

Many mothers interviewed in this study are suspicious of the MMR vaccine. One of the reasons for this is that the MMR is provided at the time children usually develop speech. Therefore, when parents see or hear that other children have stopped speaking after receiving the vaccine, they assume that it is a MMR vaccine side effect and a sign of autism. Even though some mothers already know that this is not true, they don’t want to take the risk of living with permanent guilt if their children end up being autistic. It is the fear of this risk that has compelled them to rather delay the vaccination until an age they believe is safe.

The peer pressure not to vaccinate at 18 months from other members of the community was strong and drove many to delay the vaccination. This is consistent with results from Downs et. al. who found that even the parents who favoured vaccination would be confused by ongoing debates, which would make them question their choices [14]. In this study, many mothers had simple and unchallenged beliefs about the vaccine, which could make them more vulnerable to anti-vaccination information. The negative impact that the social network can have on parents has been highlighted in other studies [11]. The mothers in the current study reported to the CHC that their friends and relatives had informed them about the alleged MMR side effects and this greatly influenced their decision to delay.

This study highlights that mothers who chose not to vaccinate had a greater fear of autism than measles, mumps and rubella. Unlike in previous studies in which Somali mothers expressed greater anxiety about their children catching infectious diseases [27], for some mothers in this study the fear of autism overshadowed fear of the infectious diseases. In line with other studies, they believed that the diseases the vaccine is intended to prevent are mild and uncommon compared to the risk of autism [28]. In addition in this study we find that both advice from peer parents and trust and distrust in nurses seem to be influencing the decision to vaccinate on time or not.

Trust in the nurses was an essential element for the mothers who vaccinated their children. The parents’ reporting of positive relations with the CHC nurses highlights the important role the nurses play in the parents’ decision to vaccinate [29]. Trust in healthcare providers and the information they provide facilitates the decision to vaccinate. Parents who vaccinated their children on time had the universal understanding that vaccines were beneficial to their children’s health and they trusted nurses, whom they regarded as being responsible for their children’s health.

The safety of the MMR vaccine has been amply tested and demonstrated [30] and its association with autism dispelled [31,32]. There is no evidence that the risk of autism is higher in children vaccinated with MMR [33], yet the rumours questioning its safety continue. The mothers interviewed in this study found the process of deciding whether to have their children vaccinated difficult and stressful because of the ongoing debate, as expressed in other studies [34]. Interventions should therefore focus on communication mechanisms and decision-making pathways that would contribute to improving immunization coverage with the MMR vaccination in this target population.

### Methodological Considerations

Several steps were taken to ensure trustworthiness of the information. Interviews were conducted in the participants language of choice and tape-recorded to capture as much detail as possible and allow for an engaging discussion. Individual interviews were conducted with a female interviewer to permit open discussion and room for more probing for deeper understanding. Triangulation was done in analysing data and sharing the preliminary findings with child health experts to increase the credibility of the data. A member check with the mothers was also conducted. In this study, all parents who did not vaccinate described themselves as delayers and not decliners and this made it difficult to identify those who declined entirely [35,36]. Great efforts were made by the research team to contact potential participants through gatekeepers. They presented the project at different venues such as local NGO meetings and open day care and used the snowball method to recruit additional participants. In this study all participants who volunteered to be interviewed were either parents who vaccinate on time and those who delayed to vaccinate on time. Therefore, we do not have the views and experiences of parents who reject the MMR vaccine, which may have re-enforced our study findings or introduced new findings.

## 5. Conclusions

Fear that the MMR vaccine can cause autism persists among Somali mothers in Tensta and Rinkeby who participated in this study and is fueled by rumours spread by their friends and relatives. Relieving this source of anxiety among the mothers should be the cornerstone of interventions by MMR vaccination programmes. The positive attitude of CHC nurses can compel mothers to vaccinate their children, due to the strong trust accorded to them. CHC nurses are the first contact between mothers and the primary health care system and this could be a strong avenue through which to improve vaccination coverage. This study informs and suggests some opportunities for tailored interventions for improving vaccination coverage and ultimately eliminating measles and rubella.

## References

[1] Bloom B.R., Marcuse E., Mnookin S. (2014). Addressing vaccine hesitancy. Science.

[2] MacDonald N.E. (2015). SAGE Working Group on Vaccine Hesitancy. Vaccine hesitancy: Definition, scope and determinants. Vaccine.

[3] Larson H.J., de Figueiredo A., Xiahong Z., Schulz W.S., Verger P., Johnston I.G., Cook A.R., Jones N.S. (2016). The State of Vaccine Confidence 2016: Global Insights Through a 67-Country Survey. EBioMedicine.

[4] Gilkey M.B., McRee A.L., Brewer N.T. (2013). Forgone vaccination during childhood and adolescence: Findings of a statewide survey of parents. Prev. Med..

[5] Gowda C., Schaffer S.E., Kopec K., Markel A., Dempsey A.F. (2013). Does the relative importance of MMR vaccine concerns differ by degree of parental vaccine hesitancy?: An exploratory study. Hum. Vaccines Immunother..

[6] Luthy K.E., Beckstrand R.L., Callister L.C. (2010). Parental hesitation in immunizing children in Utah. Public Health Nurs..

[7] Frew P.M., Hixson B., del Rio C., Esteves-Jaramillo A., Omer S.B. (2011). Acceptance of pandemic 2009 influenza A (H1N1) vaccine in a minority population: Determinants and potential points of intervention. Pediatrics.

[8] Larson H.J., Jarrett C., Eckersberger E., Smith D.M., Paterson P. (2014). Understanding vaccine hesitancy around vaccines and vaccination from a global perspective: A systematic review of published literature, 2007–2012. Vaccine.

[9] Hickler B., Guirguis S., Obregon R. (2015). Vaccine Special Issue on Vaccine Hesitancy. Vaccine.

[10] WHO (2014). SAGE Working Group on Vaccine Hesitancy.

[11] Brunson E.K. (2013). The impact of social networks on parents’ vaccination decisions. Pediatrics.

[12] Brunson E.K. (2013). How parents make decisions about their children’s vaccinations. Vaccine.

[13] Casiday R., Cresswell T., Wilson D., Panter-Brick C. (2006). A survey of UK parental attitudes to the MMR vaccine and trust in medical authority. Vaccine.

[14] Downs J.S., de Bruin W.B., Fischhoff B. (2008). Parents’ vaccination comprehension and decisions. Vaccine.

[15] Brown K.F., Shanley R., Cowley N.A., van Wijgerden J., Toff P., Falconer M., Ramsay M., Hudson M.J., Green J., Vincent C.A. (2011). Attitudinal and demographic predictors of measles, mumps and rubella (MMR) vaccine acceptance: Development and validation of an evidence-based measurement instrument. Vaccine.

[16] Bystrom E., Lindstrand A., Likhite N., Butler R., Emmelin M. (2014). Parental attitudes and decision-making regarding MMR vaccination in an anthroposophic community in Sweden—A qualitative study. Vaccine.

[17] Woudenberg T., van Binnendijk R.S., Sanders E.A., Wallinga J., de Melker H.E., Ruijs W.L., Hahne S.J. (2017). Large measles epidemic in the Netherlands, May 2013 to March 2014: Changing epidemiology. Eurosurveill.

[18] Tomlinson N., Redwood S. (2013). Health beliefs about preschool immunisations: An exploration of the views of Somali women resident in the UK. Divers. Equal. Health Care.

[19] Bahta L., Ashkir A. (2015). Addressing MMR Vaccine Resistance in Minnesota’s Somali Community. Minn. Med..

[20] Folkhälsomyndigheten (2014). Barriers Motivating Factors MMR Vaccination Communities Low Coverage Sweden.

[21] Kulane A.J.A., Robleh I., Bågenholm G. (2007). Somali Parents’ Acceptance of MMR Vaccinations for Their Children. An Exploratory Study.

[22] Butler R., MacDonald N.E., SAGE Working Group on Vaccine Hesitancy (2015). Diagnosing the determinants of vaccine hesitancy in specific subgroups: The Guide to Tailoring Immunization Programmes (TIP). Vaccine.

[23] Holloway I. (2005). Qualitative Research in Health Care.

[24] Boyce C., Neale P. (2006). Conducting in-Depth Interviews: A Guide for Designing and Conducting in-Depth Interviews for Evaluation Input.

[25] Graneheim U.H., Lundman B. (2004). Qualitative content analysis in nursing research: Concepts, procedures and measures to achieve trustworthiness. Nurse Educ. Today.

[26] Dahlgren L., Emmelin M., Winkvist A. (2007). Qualitative Methodology for International Public Health.

[27] Condon L. (2002). Maternal attitudes to preschool immunisations among ethnic minority groups. Health Educ. J..

[28] Brown K.F., Kroll J.S., Hudson M.J., Ramsay M., Green J., Long S.J., Vincent C.A., Fraser G., Sevdalis N. (2010). Factors underlying parental decisions about combination childhood vaccinations including MMR: A systematic review. Vaccine.

[29] Levi B.H. (2007). Addressing parents’ concerns about childhood immunizations: A tutorial for primary care providers. Pediatrics.

[30] Institute of Medicine Immunization Safety Review C., Stratton K., Gable A., Shetty P., McCormick M. (2001). Immunization Safety Review: Measles Mumps Rubella Vaccine and Autism.

[31] Maglione M.A., Das L., Raaen L., Smith A., Chari R., Newberry S., Shanman R., Perry T., Goetz M.B., Gidengil C. (2014). Safety of vaccines used for routine immunization of U.S. children: A systematic review. Pediatrics.

[32] Hensley E., Briars L. (2010). Closer look at autism and the measles-mumps-rubella vaccine. Am. Pharm. Assoc. JAPhA.

[33] Miller E. (2003). Measles-mumps-rubella vaccine and the development of autism. Semin. Pediatr. Infect. Dis..

[34] Evans M., Stoddart H., Condon L., Freeman E., Grizzell M., Mullen R. (2001). Parents’ perspectives on the MMR immunisation: A focus group study. Br. J. Gen. Pract..

[35] Holloway I., Todres L. (2003). The status of method: Flexibility, consistency and coherence. Qual. Res..

[36] Golafshani N. (2003). Understanding reliability and validity in qualitative research. Qual. Rep..

